# Autoreactive Plasmablasts After B Cell Depletion With Rituximab and Relapses in Antineutrophil Cytoplasmic Antibody–Associated Vasculitis

**DOI:** 10.1002/art.42388

**Published:** 2023-03-15

**Authors:** Alvise Berti, Sophie Hillion, Maximilian F. Konig, Marta Casal Moura, Amber M. Hummel, Eva Carmona, Tobias Peikert, Fernando C. Fervenza, Cees G. M. Kallenberg, Carol A. Langford, Peter A. Merkel, Paul A. Monach, Philip Seo, Robert F. Spiera, Paul Brunetta, E. William Clair, Kristina M. Harris, John H. Stone, Guido Grandi, Jacques-Olivier Pers, Ulrich Specks, Divi Cornec

**Affiliations:** 1 Division of Pulmonary & Critical Care Medicine, Thoracic Disease Research Unit, Mayo Clinic, Rochester, Minnesota, and Center for Medical Sciences (CISMed), Department of Cellular, Computational and Integrative Biology (CIBIO), University of Trento, Italy, and Rheumatology Unit, Santa Chiara Hospital, APSS Trento, Italy; 2 Jacques-Olivier Pers, DDS, PhD, Divi Cornec, MD, PhD: Université de Bretagne Occidendale, Brest, Bretagne, France; 3 Division of Rheumatology, Department of Medicine, The Johns Hopkins University School of Medicine, Baltimore, Maryland; 4 Division of Pulmonary & Critical Care Medicine, Thoracic Disease Research Unit, Mayo Clinic, Rochester, Minnesota; 5 Division of Nephrology & Hypertension, Mayo Clinic, Rochester, Minnesota; 6 Department of Rheumatology and Clinical Immunology, University of Groningen, Groningen, The Netherlands; 7 Cleveland Clinic, Cleveland, Ohio; 8 Division of Rheumatology, Department of Medicine, and Department of Biostatistics, Epidemiology, and Informatics, Division of Clinical Epidemiology, University of Pennsylvania, Philadelphia; 9 Brigham and Women’s Hospital, Boston, Massachusetts; 10 Weill Cornell Medical College, Hospital for Special Surgery, New York; 11 Genentech, Inc., San Francisco, California; 12 Duke University, Durham, North Carolina; 13 Immune Tolerance Network, Bethesda, Maryland; 14 Massachusetts General Hospital Rheumatology Unit, Boston; 15 Department of Cellular, Computational and Integrative Biology (CIBIO), University of Trento, Italy.

## Abstract

**Objective.:**

Autoreactive B cells are responsible for antineutrophil cytoplasmic antibody (ANCA) production in ANCA-associated vasculitis (AAV). Rituximab (RTX) depletes circulating B cells, including autoreactive B cells. We aimed to evaluate changes and associations with relapse of the circulating autoreactive B cell pool following therapeutic B cell depletion in AAV.

**Methods.:**

Sequential flow cytometry was performed on 148 samples of peripheral blood mononuclear cells from 23 patients with proteinase 3 (PR3)–ANCA–positive AAV who were treated with RTX for remission induction and monitored after stopping therapy during long-term follow-up in a prospective clinical trial. PR3 was used as a ligand to target autoreactive PR3-specific (PR3+) B cells. B cell recurrence was considered as the first blood sample with ≥10 B cells/μl after RTX treatment.

**Results.:**

At B cell recurrence, PR3+ B cell frequency among B cells was higher than baseline (*P* < 0.01). Within both PR3+ and total B cells, frequencies of transitional and naive subsets were higher at B cell recurrence than at baseline, while memory subsets were lower (*P* < 0.001 for all comparisons). At B cell recurrence, frequencies of B cells and subsets did not differ between patients who experienced relapse and patients who remained in remission. In contrast, the plasmablast frequency within the PR3+ B cell pool was higher in patients who experienced relapse and associated with a shorter time to relapse. Frequencies of PR3+ plasmablasts higher than baseline were more likely to be found in patients who experienced relapse within the following 12 months compared to those in sustained remission (*P* < 0.05).

**Conclusion.:**

The composition of the autoreactive B cell pool varies significantly following RTX treatment in AAV, and early plasmablast enrichment within the autoreactive pool is associated with future relapses.

## INTRODUCTION

The antineutrophil cytoplasmic antibody (ANCA)–associated vasculitides, which include ANCA–associated vasculitis (AAV), are a group of systemic autoimmune diseases characterized by the presence of autoantibodies directed against proteinase 3 (PR3) or myeloperoxidase (MPO) (1–3) in the serum of most patients. In AAV, B cells are central in the development of the disease and the production of ANCAs (4,5), which mediate the disease by a variety of proinflammatory mechanisms, including the triggering of neutrophil activation and degranulation (6,7). ANCA levels are weakly associated with disease activity (8–10), and this association is affected by disease phenotype and remission induction treatment, particularly in patients presenting with renal involvement, alveolar hemorrhage, and severe relapses (11).

Rituximab (RTX), which induces B cell depletion by targeting CD20 on the B cell surface, has become a standard treatment option for AAV based on the results of 2 randomized controlled clinical trials (12–14), and the short- and long-term efficacy of RTX to control disease activity in patients with AAV has been confirmed in subsequent cohort studies and remission maintenance trials (15–20).

Autoreactive B cells are responsible for autoantibody production in autoimmune diseases, such as antinuclear antibodies in lupus and anti–citrullinated protein antibodies in rheumatoid arthritis (21–25). Similarly, autoreactive B cells are responsible for ANCA production in AAV (26). The existence of circulating B cells bearing a B cell receptor specific for PR3 or MPO has been postulated for years, but reliable detection of these cells has been elusive until recently (27,28). We previously developed a customized flow cytometry method to evaluate circulating autoreactive PR3-specific (PR3+) B cells among cryopreserved peripheral blood mononuclear cells (PBMCs) (27). We were able to characterize the phenotype and the function of PR3+ B cells in patients with PR3-AAV and healthy controls (27). However, after RTX-induced depletion of B cells, the reconstitution and subsequent persistence of circulating PR3+ B cells, their repartition between the different B cell subsets within the PR3+ pool, and their relationship with long-term treatment outcomes remain unknown.

Using this flow cytometry–based assay, we investigated the longitudinal changes of circulating PR3+ B cells in PBMCs from 23 patients with severe PR3-ANCA–positive AAV who had achieved complete remission with RTX and glucocorticoids within 6 months after initiation of remission induction therapy in a clinical trial (12). For this proof-of-concept study, we hypothesized that RTX-induced B cell depletion would alter the proportions of circulating B cell subsets within the autoreactive pool and investigated whether any features of these changes during follow-up were associated with relapse.

## PATIENTS AND METHODS

### Study population and design.

Twenty-three patients with PR3-ANCA–positive AAV from the RTX treatment group of the RAVE trial (ClinicalTrials.gov identifier NCT00104299) (12) who reached the primary end point of the study (Birmingham Vasculitis Activity Score for Wegener’s Granulomatosis of 0 and not receiving glucocorticoids at month 6) with available baseline and follow-up PBMC samples were selected for our analysis and provided 148 unique serial PBMC samples. PBMCs had been collected and cryopreserved upon enrollment, at month 6, and every 3 months until month 18, then annually after month 18, according to the trial protocol (13) (see Supplementary Methods 1, available on the *Arthritis & Rheumatology* website at https://onlinelibrary.wiley.com/doi/10.1002/art.42388).

According to the trial’s definition, B cell recurrence (or redetection) in RTX-treated trial participants was defined as ≥10 but <69 CD19+ cells/μl, and reconstitution was defined as ≥69 CD19+ cells/μl or a return to baseline levels. All clinical data were obtained from the trial database.

To ensure comparability of the subsets of B cells and autoreactive cells between baseline and B cell recurrence (i.e., assuring a minimum number of B cells at B cell recurrence to allow for an accurate assessment of B cell subsets), we considered the biologic time point of B cell recurrence as the first blood sample at or after month 6 in which study participants had ≥10 B cells/μl.

### Recombinant PR3 production and labeling.

A recombinant PR3 (rPR3) was expressed in an epithelial cell line and labeled as previously described (27–29) (see Supplementary Methods 2 at https://onlinelibrary.wiley.com/doi/10.1002/art.42388).

### ANCA testing.

PR3-ANCA IgG levels were determined by enzyme-linked immunosorbent assay (ELISA) (Euroimmun) in all serum samples from all 23 patients as previously described (11).

### PR3-reactive B cell detection and fluorescence-activated cell sorting (FACS) analysis.

PBMCs frozen in 20% DMSO/human AB serum were thawed, counted, and stained for flow cytometric analysis. Labeled PR3 was used as a ligand to target autoreactive B cells. In brief, 1 × 10^6^ cells were incubated on ice for 20 minutes with biotin-labeled rPR3 and a cocktail of antibodies (allophycocyanin [APC]/Alexa Fluor 700–conjugated anti-CD19, APC-conjugated anti-human IgD, PC7-conjugated anti-CD27, PC5.5-conjugated anti-CD38, and APC/Alexa Fluor 750–conjugated anti-CD24; all from Beckman Coulter), washed 3 times, incubated for 15 minutes with streptavidin–fluorescein isothiocyanate, washed, and fixed. For each experiment, unstained cells as well as single-color controls were included. Cell analysis was performed using a FACSCanto system (BD Bioscience), as previously described (27). FACS data were analyzed and graphed using Kaluza version 1.5a (Beckman Coulter) and FlowJo software version 10.

### Functional validation of the PR3+ B cell FACS method.

We studied whether PR3+ B cells identified by flow cytometry could secrete PR3-ANCA. PBMCs of patients with active PR3-AAV were sorted by FACS based on streptavidin expression to isolate PR3+CD19+ and PR3–CD19+ cells. Sorted B cells were cultured and stimulated to promote B cell activation using modifications of previously published protocols (30,31) (see Supplementary Methods 3 at https://onlinelibrary.wiley.com/doi/10.1002/art.42388). To validate PR3-ANCA secretion at the level of a single B cell, peripheral blood B cells from a patient with severe, untreated PR3-AAV were immortalized using Epstein-Barr virus to obtain lymphoblastoid cell lines (32), and PR3+ and PR3– cells were sorted for single-cell culture. Cell culture supernatants of PR3+ and PR3– lymphoblastoid cell line single-cell clones were analyzed by ELISA to quantify anti-PR3 antibodies (IgM or IgG isotype) (see Supplementary Methods 4, https://onlinelibrary.wiley.com/doi/10.1002/art.42388).

### Statistical analysis.

Categorical data are presented as the number (percentage), and continuous data are presented as the median (range or interquartile range [IQR]) or mean ± SEM. Groups were compared using parametric or nonparametric tests when appropriate, and Student’s *t*-test or Mann-Whitney test was used for continuous data. Wilcoxon’s signed rank test was used to analyze paired data before and after RTX treatment, and chi-square test or Fisher’s exact test, as appropriate, was used for categorical data.

Clinical outcomes were correlated with B cells and PR3+ B cells and their subsets. The estimated distributions of relapse and severe relapse were performed with the Kaplan-Meier method and the log rank test.

To further assess the ability of using the frequency of plasmablasts (PBs) within the PR3+ B cell pool to distinguish between patients who experience relapse and patients who remain in long-term remission, a receiver operating characteristic (ROC) curve was constructed using logistic regression, with the percentage of the circulating biomarker as the predictor variable and relapse versus remission during follow-up as the dichotomous outcome. The area under the ROC curve (AUC or C statistic) was calculated.

To assess for a possible association between an increase of peripheral blood PBs among the PR3+ B cell pool (PR3+ PBs) and relapse, we performed a case-time-control analysis, which comprises a regular case crossover of cases (relapse) and a case crossover of controls (sustained remission). For this analysis, 148 samples (all available samples from the 23 included patients) were used. For each individual study participant, the increase in frequency of PR3+ PBs after B cell depletion was defined as a PR3+ PB level during follow-up equal to or higher than the individual’s baseline level. For the patient to be considered at risk (“exposed”), the increase in PR3+ PB level had to occur within the 12 months preceding the relapse. To calculate the effect of an increase in PR3+ PBs, we used conditional logistic regression. The ratio between the odds ratio (OR) from the case group of the study and the OR obtained from the control group generated the case-time-control study OR risk for relapse if an increase in PR3+ PBs occurred.

A vector graphic editing program was used to build the swimmer plot (Affinity Designer; Serif). Conditional logistic regression was calculated using Stata version 13.1 (StataCorp). All other statistical analyses were performed using JMP version 8 (SAS Institute) or GraphPad Prism software version 8.

## RESULTS

### Remission, relapse, and B cell recurrence in PR3-AAV.

**Figure 1.**
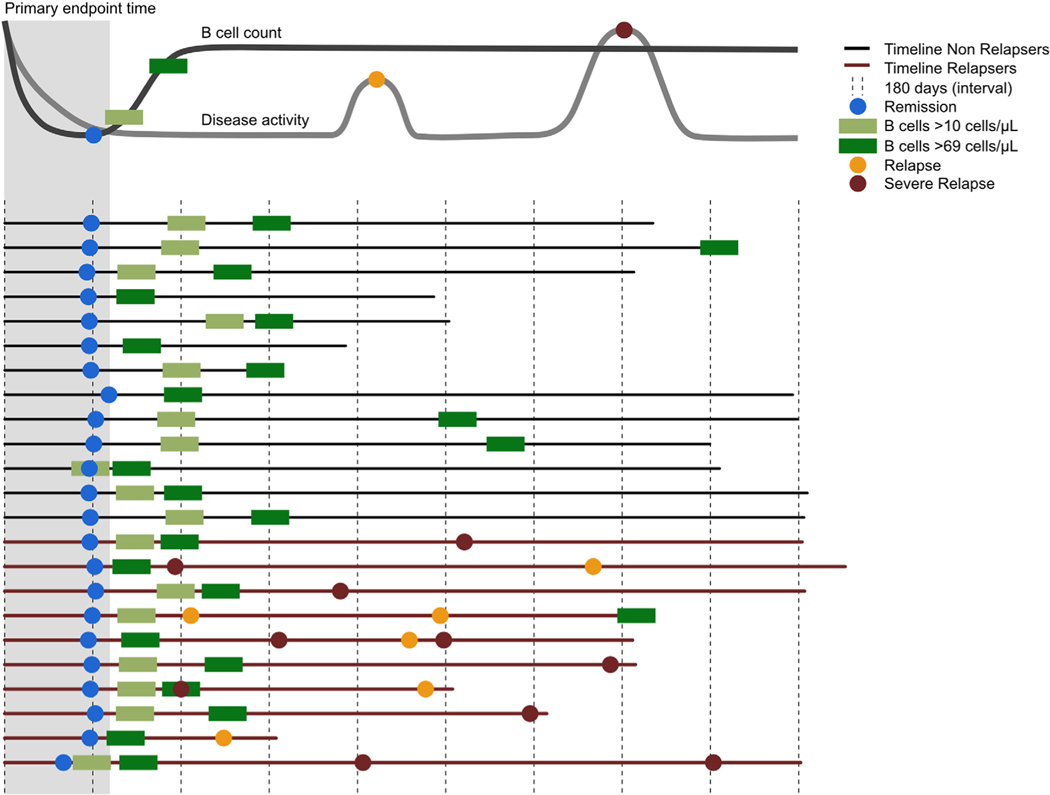
Swimmer plot showing response to rituximab (RTX) treatment, relapse status, and B cell dynamics during follow-up in 23 patients with proteinase 3–antineutrophil cytoplasmic antibody–associated vasculitis. Timelines represent individual patients. After induction of remission with RTX and glucocorticoids, complete remission while no longer receiving RTX or glucocorticoid therapy was achieved by all patients before month 6 after randomization. Ten patients experienced relapse during follow-up, of whom 8 patients had severe relapse, 5 patients had nonsevere relapse, and 5 patients had multiple relapses during the observation period. All the patients repopulated B cells during follow-up, and no patient experienced relapse before reaching a B cell count >69 cells/μl.

Clinical and demographic features of the study participants are reported in Supplementary Table 1, available at https://onlinelibrary.wiley.com/doi/10.1002/art.42388. All patients were treated with RTX and glucocorticoids for the induction of remission, achieved complete remission within 6 months after randomization, and were not receiving RTX or glucocorticoid treatment after month 6 unless a relapse occurred (Figure 1). Median follow-up was 44 months (25–75% IQR 31–54 months) among all included patients, 43 months (25–75% IQR 35–54 months) for patients who experienced a relapse after achieving complete remission, and 48 months (25–75% IQR 30–54 months) for patients who remained in complete remission for the duration of the trial (*P* = 0.975). Ten patients experienced a relapse during follow-up, of whom 8 patients had severe relapses; 5 patients had multiple relapses during the observation period (Figure 1).

**Figure 2.**
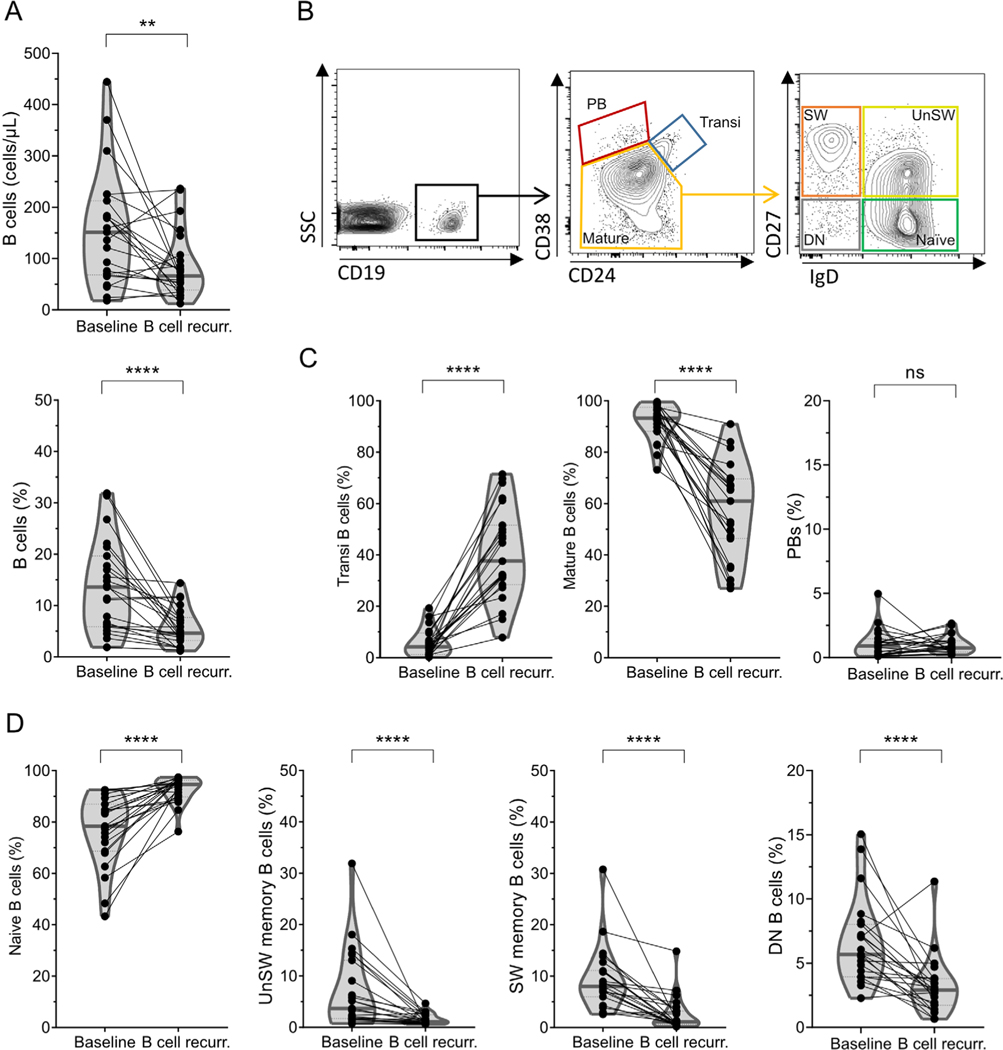
Pairwise comparisons between baseline and B cell recurrence (recurr) of the count (**A**, top) and frequency (**A**, bottom) of B cells among cryopreserved peripheral blood mononuclear cells obtained from patients with proteinase 3–antineutrophil cytoplasmic antibody–associated vasculitis who achieved remission after therapeutic B cell depletion with rituximab. **B**, Gating strategy for plasmablast (PB), transitional (Transi), and mature switched (SW), unswitched (UnSW), double negative (DN), and naive B cell subsets. **C** and **D**, Pairwise comparisons of the frequencies of B cell subsets within circulating B cells between baseline and B cell recurrence. At B cell recurrence, a median of 37.56% (25–75% interquartile range [IQR] 28.35–51.56%) of circulating B cells expressed a transitional phenotype. Plasmablasts (CD19+CD24–CD38^high^) did not significantly change from baseline (**C**). Within the mature B cell pool (CD19+CD24^low/high^CD38^low^), the frequency of naive (CD27–IgD+) B cells was significantly higher, and the frequencies of unswitched (CD27+IgD+), switched (CD27+IgD–), and double negative (CD27–IgD–) memory subsets were significantly lower at B cell recurrence compared to baseline (**D**). Symbols represent individual patient samples. Thick horizontal lines show the median; dotted horizontal lines show the IQR. ** = *P* < 0.01 and **** = *P* < 0.0001. ns = not significant. Color figure can be viewed in the online issue, which is available at http://onlinelibrary.wiley.com/doi/10.1002/art.42388/abstract.

We compared cryopreserved PBMCs collected at baseline with those collected at B cell recurrence. The median time point of B cell recurrence was month 12 (range 6–24). At B cell recurrence, absolute B cell counts and relative frequencies were significantly lower compared to baseline (Figure 2A). Among B cell subsets, the frequencies of transitional (CD19+CD24^high^CD38^high^) and naive B cells were significantly higher, while the frequencies of the memory B cell subsets were significantly lower at B cell recurrence than at baseline. The gating strategy and frequencies of B cell subsets are shown in Figures 2B–D and Supplementary Figure 1, https://onlinelibrary.wiley.com/doi/10.1002/art.42388.

### Identification and validation of the PR3-reactive B cells by FACS.

Upon stimulation in culture, the staining of cytospinned B cells showed signs of activation and initial cytoplasmic IgG accumulation, suggestive of an initial differentiation of memory B cells toward antibody-secreting cells in both PR3+CD19+ and PR3–CD19+ sorted cells (Figures 2A and B). PR3-ANCA IgG was detected by ELISA only in the supernatant of PR3+CD19+ cell cultures and not of PR3–CD19+ cell cultures or in other negative controls (RPMI growth medium, supernatant of 4-day stimulated CD19+ B cell cultures from healthy controls, or purified MPO-ANCA IgG from the PR3-AAV patients) (Figure 2B), indicating that our detection methodology ensured a full recovery of circulating PR3-reactive B cells within the PR3+ B cell pool as detected by FACS.

**Figure 3.**
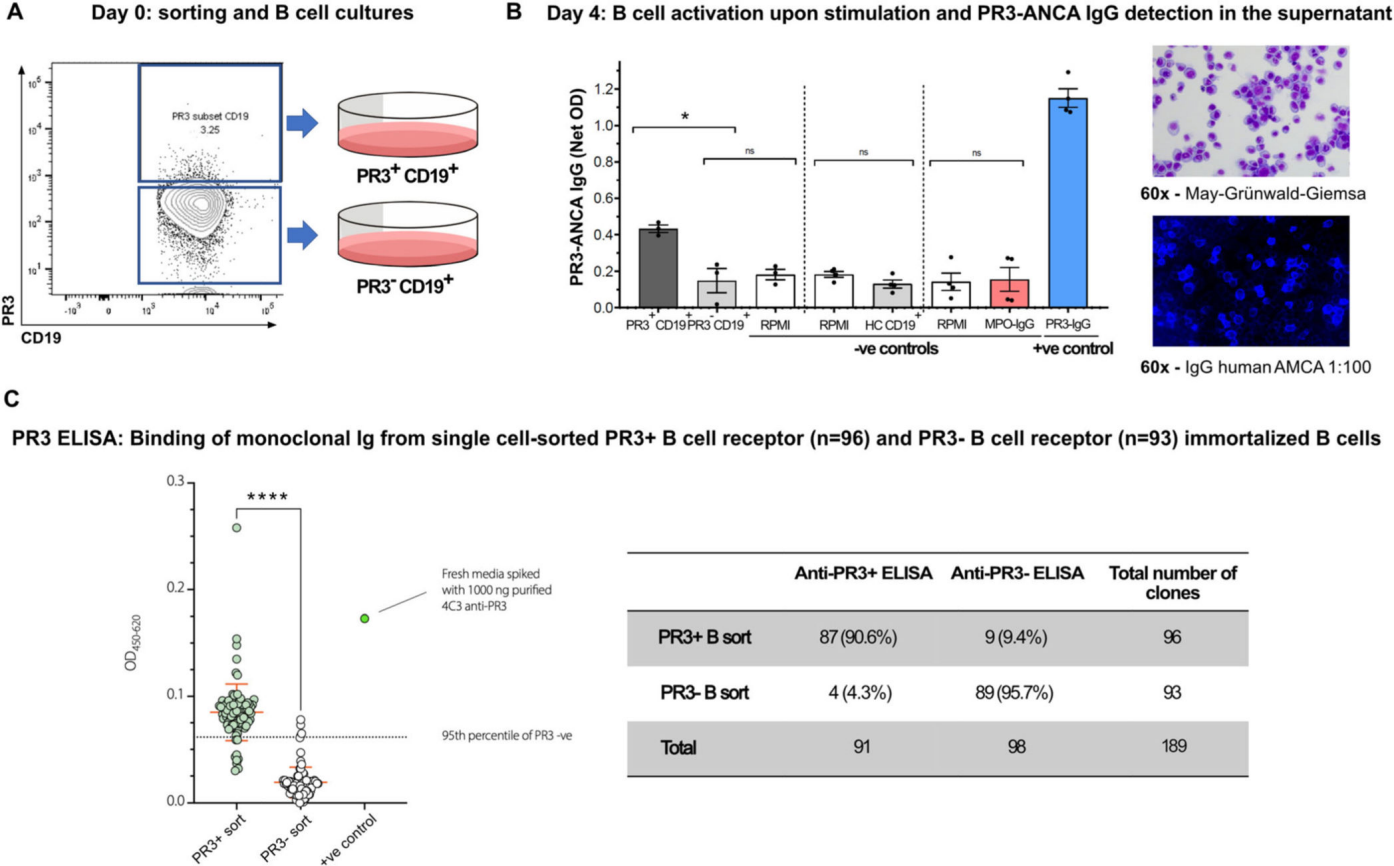
Functional validation of autoreactive proteinase 3–specific (PR3+) B cells by sorting and culturing PR3+ and PR3– B cells (**A**). **B**, Left, PR3–antineutrophil cytoplasmic antibody (ANCA) IgG measured using enzyme-linked immunosorbent assay (ELISA) after 4-day culture of bulk-sorted PR3+ and PR3– B cells from 3 PR3-ANCA–associated vasculitis (PR3-AAV) patients compared to B cells from 4 healthy controls (HCs) and purified myeloperoxidase (MPO)–ANCA and PR3-ANCA from 4 patients as negative (-ve) and positive (+ve) controls. **B**, Right, Representative staining of cytospin smears at day 4 with May-Grünwald-Giemsa stain to assess B cell activation and with aminomethylcoumarin acetate (AMCA)–conjugated anti-human IgG in immunofluorescence to assess initial cytoplasmic IgG accumulation. **C**, PR3-specific Ig secretion as measured by PR3-specific ELISA in cell culture supernatants (at week 4) of single cell–sorted PR3+ and PR3– Epstein-Barr virus–immortalized B cells from a patient with severe, untreated PR3-AAV. Native PR3 ELISA was used to measure phenylmethylsulfonyl fluoride–treated anti-human IgG and IgM. Cutoff for positivity was defined as the 95th percentile of PR3– B cells. Recombinant patient-derived IgG anti-PR3 is shown as positive control. In **B**, symbols represent individual samples. Bars with whiskers show the mean ± SEM net OD. In **C**, horizontal lines with whiskers show the mean ± SD background-corrected OD. * = *P* < 0.05 and **** = *P* < 0.0001. ns = not significant. Color figure can be viewed in the online issue, which is available at http://onlinelibrary.wiley.com/doi/10.1002/art.42388/abstract.

We then analyzed the binding of 189 monoclonal antibodies from the supernatants of single-sorted immortalized B cells from a patient with active severe PR3-AAV as detected by PR3-specific ELISA, showing that 91% of the sorted PR3+ B cells (n = 96) had detectable PR3-specific antibodies in the supernatants (i.e., true positive), while 96% of the sorted PR3– B cells (n = 93) did not have detectable antibodies against PR3 (i.e., true negative) (Figure 3C). This validated our customized FACS method, demonstrating that the great majority of the PR3+ B cells identified in patients’ blood are truly PR3-specific clones.

### Recurrence and subset redistribution of circulating autoreactive B cells following RTX-induced remission in PR3-AAV.

**Figure 4.**
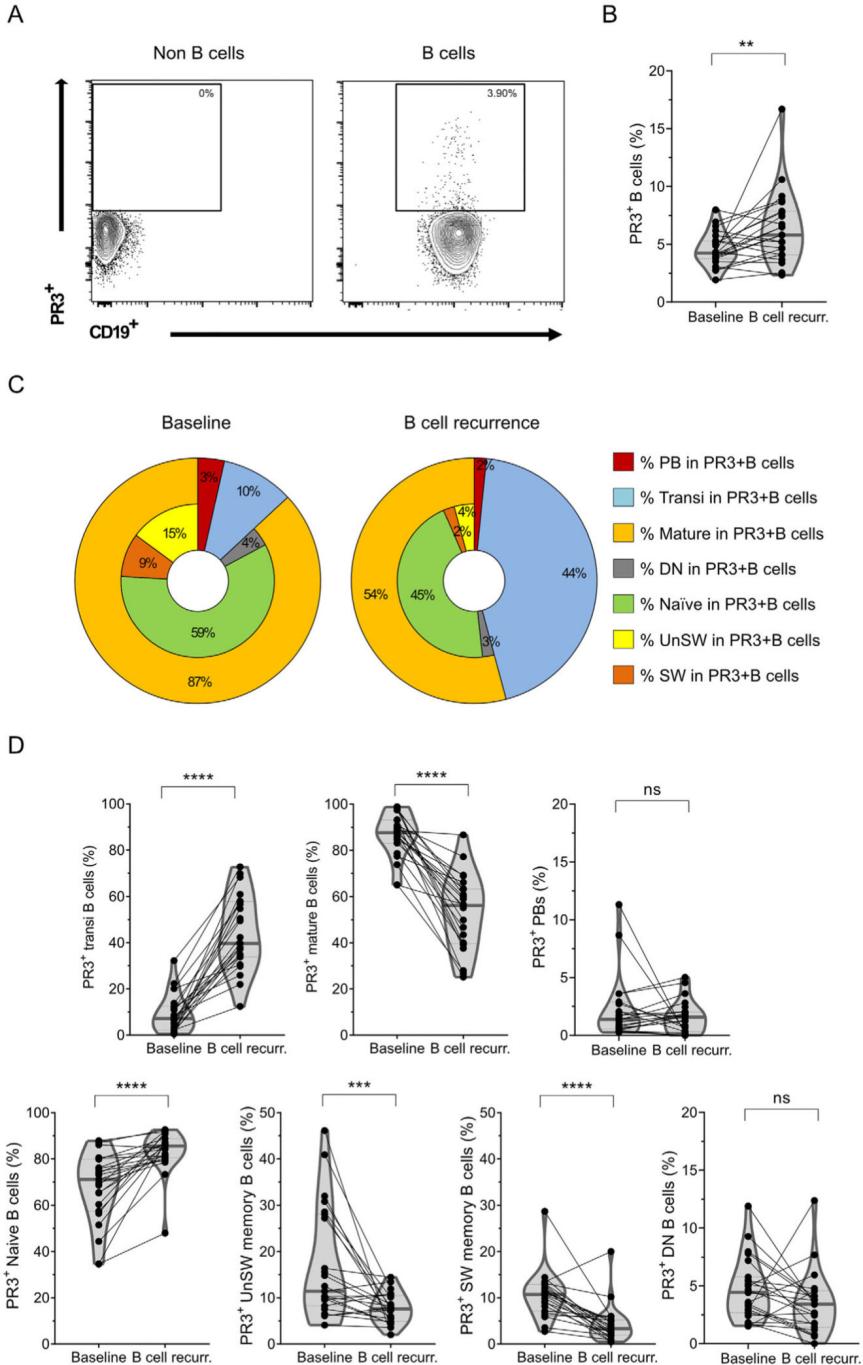
Recurrence and redistribution of circulating autoreactive B cell subsets following therapeutic B cell depletion with rituximab in patients with proteinase 3–antineutrophil cytoplasmic antibody–associated vasculitis. **A**, Gating strategy for autoreactive B cell subset. **B**, Pairwise comparison of the frequency of proteinase 3–specific (PR3+) B cells between baseline and B cell recurrence. **C**, Frequencies of B cell subsets within the PR3+ B cell pool at baseline and at B cell recurrence. **D**, Pairwise comparisons of circulating PR3+ B cell subsets between baseline and B cell recurrence. In **B** and **D**, symbols represent individual patient samples. Thick horizontal lines show the median; dotted horizontal lines show the IQR. ** = *P* < 0.01; *** = *P* < 0.001; **** = *P* < 0.0001. See Figure 2 for other definitions. Color figure can be viewed in the online issue, which is available at http://onlinelibrary.wiley.com/doi/10.1002/art.42388/abstract.

The frequency of circulating PR3+ B cells was significantly higher at B cell recurrence compared to baseline (median 5.82% [25–75% IQR 4.11–7.87%] versus 4.25% [25–75% IQR 3.77–5.30%], respectively; *P* = 0.025) (see gating strategy in Figure 4A and frequencies of PR3+ B cells in Figure 4B). Similar to what was observed for total B cells, the composition of the B cell subsets within the PR3+ reactive pool at B cell recurrence was substantially different from baseline, with an increase in frequency of transitional subsets (*P* < 0.001), a decrease in frequency of mature naive subsets (*P* < 0.001), and a decrease in frequency of mature switched (*P* < 0.001) and unswitched (*P* = 0.002) memory B cell subsets, but no significant change in frequency of mature double negative or PB subsets (Figure 4C).

Pairwise comparisons showed that the frequencies of circulating transitional and naive subsets were higher, and the frequencies of mature, unswitched and switched memory subsets were lower within the autoreactive pool at B cell recurrence compared to baseline (*P* < 0.001 for all comparisons) (Figure 4D).

### Dynamics of total B cells and autoreactive B cells and relapse during long-term follow-up.

**Figure 5.**
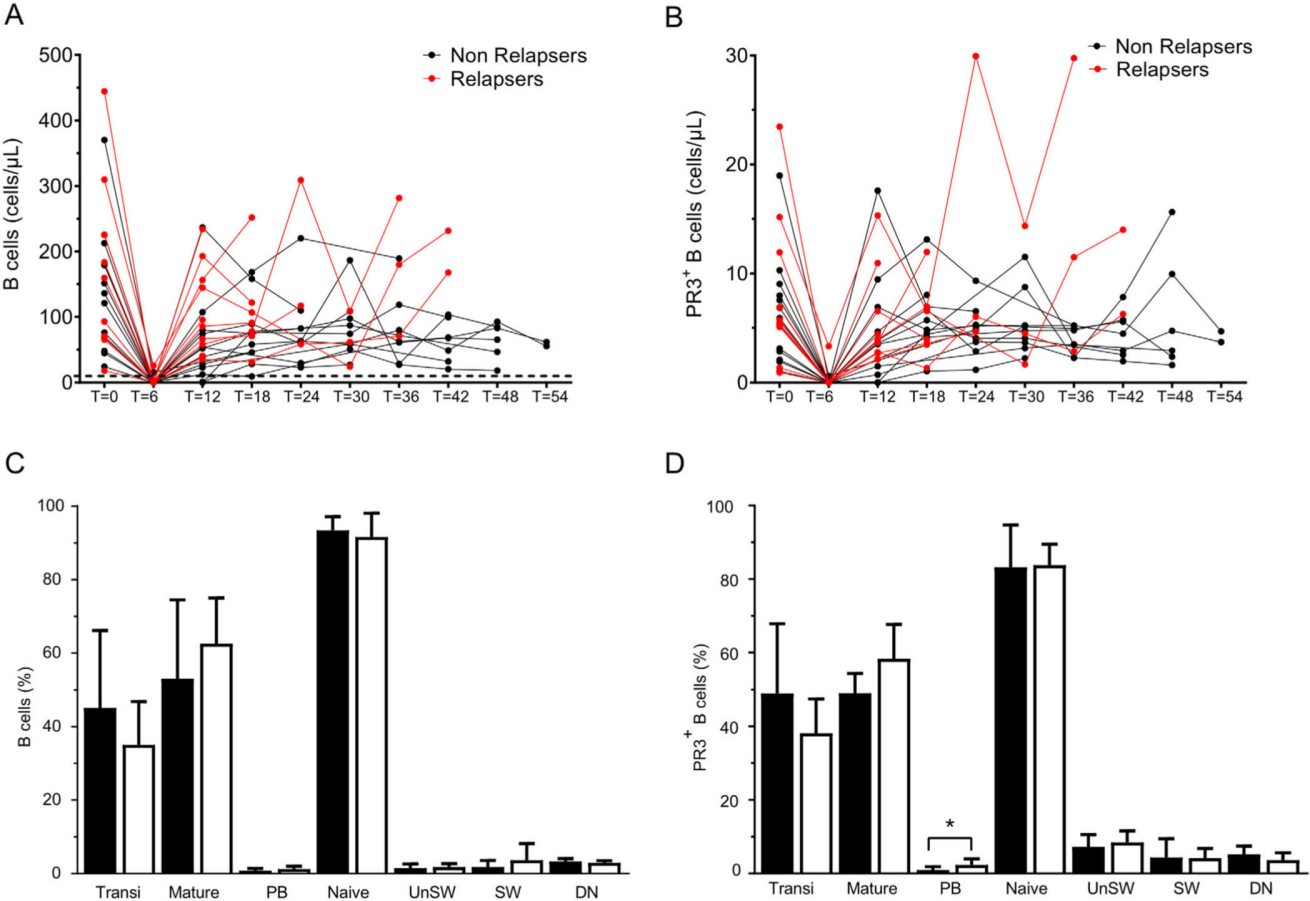
Counts of total B cells (**A**) and autoreactive proteinase 3–specific (PR3+) B cells (**B**) at baseline and during follow-up between patients with PR3–antineutrophil cytoplasmic antibody–associated vasculitis who experienced relapse and those who remained in remission after therapeutic B cell depletion with rituximab. All the patients repopulated total B cells and autoreactive B cells during follow-up; 2 patients had detectable total B cells and autoreactive B cells at month 6 (T = 6). **C** and **D**, Frequency at B cell recurrence of subsets within total B cells (**C**) and autoreactive B cells (**D**) in patients who experienced relapse (**open bars**) or who maintained long-term remission (**solid bars**). In **A** and **B**, symbols represent individual patient samples. In **C** and **D**, bars with whiskers show the mean and upper SD. * = *P* < 0.05. See Figure 2 for other definitions. Color figure can be viewed in the online issue, which is available at http://onlinelibrary.wiley.com/doi/10.1002/art.42388/abstract.

Overall, the dynamics of circulating total B cell and PR3+ B cell counts in peripheral blood of AAV patients who achieved complete remission following RTX treatment were similar between patients who experienced relapse and patients who remained in remission throughout the time points evaluated (*P* > 0.05 for all time point comparisons) (Figures 5A and B and Supplementary Figures 2A–C, https://onlinelibrary.wiley.com/doi/10.1002/art.42388). After RTX treatment, total B cells and PR3+ B cells were depleted (<10 cells/μl) in all participants (Figures 5A and B). Total B cells and PR3+ B cells repopulated during follow-up in all participants, but only 2 participants had detectable total B cells and autoreactive B cells at month 6, of whom 1 patient experienced relapse and 1 patient maintained remission throughout the follow-up period. Of note, all participants who experienced relapse had repopulated total B cells and PR3+ B cells before the clinical relapse occurred.

The composition of the peripheral blood subsets within PR3+ B cells and total B cells was similar at baseline between patients who experienced relapse and patients who remained in remission (Supplementary Figures 2C and D, https://onlinelibrary.wiley.com/doi/10.1002/art.42388). At B cell recurrence, no differences were observed between patients who experienced relapse and those who stayed in remission in the frequency of total B cells (median 5.44% [25–75% IQR 3.65–10.45%] versus 4.11% [25–75% IQR 2.30–7.17%], respectively; *P* = 0.410) or in the frequency of PR3+ B cells (median 4.65% [25–75% IQR 3.18–7.02%] versus 6.5% [25–75% IQR 5.34–8.44%], respectively; *P* = 0.121). The frequencies of all subsets evaluated within B cells at B cell recurrence were similar between these 2 disparate long-term outcome groups (Figure 5C); in particular, the frequencies of transitional and PB subsets were similar between patients who experienced relapse and patients who remained in remission. However, a significantly higher frequency of PBs was observed within the PR3+ autoreactive pool at B cell recurrence in patients who experienced relapse compared to patients who remained in remission (Figure 5D), and this subset was further enriched in patients who experienced severe relapse compared to nonsevere relapse (Supplementary Figure 2E, https://onlinelibrary.wiley.com/doi/10.1002/art.42388). Overall, enrichment in the frequency of PBs within the PR3+ pool at recurrence was the only B cell biomarker that differed between patients who experienced relapse and patients who remained in long-term remission.

### Relationship of enrichment of plasmablasts within circulating autoreactive B cells with relapse.

Since PBs were enriched within the circulating autoreactive PR3+ B cell pool in patients who experienced relapse compared to patients with long-term remission at B cell recurrence, we aimed to determine a metric of PB frequency within autoreactive B cells associated with relapse risk. The median follow-up from B cell recurrence to the last clinical evaluation was 31 months (25–75% IQR 18–42 months) among all included patients; 31 months (25–75% IQR 23–42 months) for patients who experienced relapse, and 32 months (25–75% IQR 15–40 months) for patients who remained in remission (*P* = 0.574).

**Figure 6.**
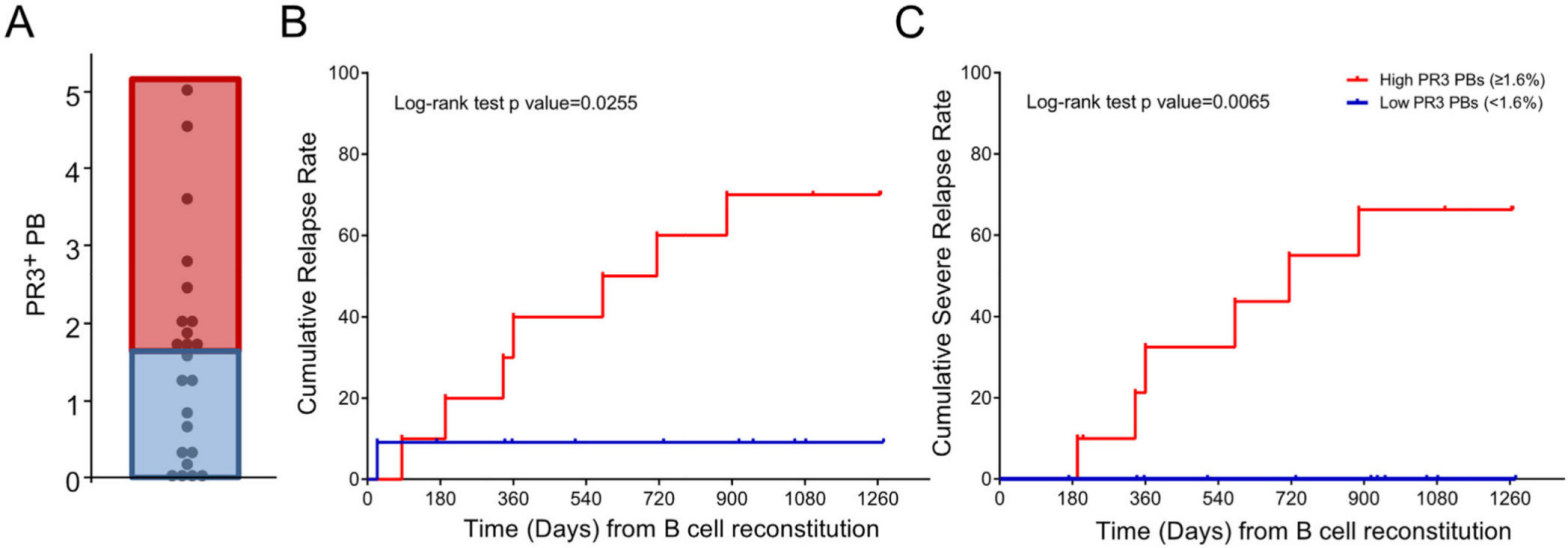
Enrichment of plasmablasts (PBs) within circulating autoreactive B cells and relationship with relapse after therapeutic B cell depletion with rituximab in patients with proteinase 3 (PR3)–antineutrophil cytoplasmic antibody–associated vasculitis. **A**, Distribution of PB frequency within the autoreactive PR3–specific (PR3+) B cell pool at B cell recurrence, showing a median value for the cohort of 1.6%. **B** and **C**, Time to relapse (**B**) and time to severe relapse (**C**) by level of PBs within the autoreactive PR3+ B cell pool. Blue shading and blue lines indicate PR3+ PB frequency < 1.6%; red shading and red lines indicate PR3+ PB frequency ≥ 1.6%. Color figure can be viewed in the online issue, which is available at http://onlinelibrary.wiley.com/doi/10.1002/art.42388/abstract.

At B cell recurrence, the frequency of PBs within the circulating autoreactive B cell pool ranged from 0.0% to 5.0%, with a median of 1.6% (Figure 6A). We hypothesized that patients with higher levels of PBs within autoreactive PR3+ B cells were at higher risk of relapse. The optimal cutoff level of PBs within autoreactive PR3+ B cells at B cell recurrence to discriminate patients who experienced relapse from those who remained in remission using an ROC curve was 1.6% (AUC 0.79, *P* = 0.013) (Supplementary Figure 3, https://onlinelibrary.wiley.com/doi/10.1002/art.42388). We observed that study participants with ≥1.6% of PBs within PR3+ B cells at B cell recurrence had a significantly shorter time to first relapse (*P* for log rank test = 0.026) and time to first severe relapse (*P* for log rank test = 0.007) (Figures 6B and C).

To explore whether an increased frequency of PBs among circulating PR3+ B cells occurs before each relapse, we performed a case-crossover analysis to evaluate if each relapse was preceded by a rise of PBs in autoreactive B cells within the previous 12 months. After B cell depletion, PR3+ PB cells were redetected in all participants at some point during the follow-up. Sixteen relapses occurred in 10 of the 23 participants studied (Figure 1). Among patients who experienced relapse, the increase of PBs within the autoreactive pool in the 12 months preceding each relapse was ~2-fold and significantly more likely to occur (OR 2.10 [95% confidence interval (95% CI) 1.07–4.11], *P* = 0.031). In contrast, the increase of PR3+ PBs among patients who remained in remission was smaller and nonsignificant (OR 1.40 [95% CI 0.76–2.59], *P* = 0.278), resulting in a case-time-control OR for the increase of autoreactive PBs of 1.50.

Since an increase of PR3-ANCA has previously been shown to precede severe relapses in patients treated with RTX (11), we repeated the previous analysis using PR3-ANCA IgG levels. At baseline, PR3-ANCA was detectable in all participants regardless of future relapse status (median 243.5 RU/ml [25–75% IQR 95.7–327 RU/ml] versus 266 RU/ml [25–75% IQR 86.35–366 RU/ml] in patients who remained in remission; *P* = 0.6418). Although PR3-ANCA levels at B cell recurrence were increased in patients who experienced relapse compared to patients who remained in remission (*P* < 0.05) (Supplementary Figure 2F, https://onlinelibrary.wiley.com/doi/10.1002/art.42388), PR3-ANCA levels higher than the median at B cell recurrence (36.1 RU/ml; range 3.4–331) were not associated with shorter time to relapse (*P* for log rank test = 0.157) or severe relapse (*P* for log rank test = 0.414) by time-to-event analyses.

Consistently, after B cell depletion, increases of PR3-ANCA levels (if the assay had previously become positive) or reappearance of PR3-ANCA (becoming detectable from undetectable levels if the assay was previously negative) did not predict PR3+ PB increase or reappearance (Supplementary Table 2, https://onlinelibrary.wiley.com/doi/10.1002/art.42388).

## DISCUSSION

This study shows a major change in the composition of B cell subsets within the circulating autoreactive pool after RTX-induced B cell depletion in patients with AAV who achieved complete remission, and this study supports the hypothesis that an early enrichment of the PB frequency within autoreactive B cells during the reconstitution process is indicative of future relapse when therapy is stopped. This finding significantly contributes to clarifying the biologic mechanisms underpinning disease relapse and prolonged remission in AAV, further providing mechanistic insights of the dynamics of B cells and autoreactive B cells in response to targeted therapy in AAV.

Specifically, we performed a detailed longitudinal analysis of autoreactive, antigen-specific PR3+ B cells before and after B cell depletion in patients with PR3-ANCA–positive AAV who were successfully treated with RTX and did not receive any other potentially confounding treatment after remission induction. We compared baseline levels of B cell subsets with those at the time point of B cell recurrence, which is characterized by an increase in the frequency of transitional B cells and a reduction in the frequency of mature B cells. The depletion of B cells after RTX treatment lasted for ~1 year in the majority of patients, and B cell counts at B cell recurrence remained lower than baseline. Clinically, the prolonged depletion of B cells after RTX treatment in AAV is well recognized (15,33), and it may affect B cell ontogeny at reconstitution, as shown by the increased proportion of peripheral blood transitional B cells and correspondingly reduced memory B cell compartments after RTX treatment in patients with rheumatoid arthritis, lupus, and pemphigus, and during reconstitution after hematopoietic stem cell transplantation (34–39). Notably, the timing of B cell repopulation following RTX therapy seems to be different in different diseases, with AAV patients experiencing reconstitution of B cells later compared to patients with rheumatoid arthritis, connective tissue diseases, and pemphigus (20,33,38).

Circulating autoreactive B cells were detectable in all the included AAV patients following B cell recurrence. The frequency of B cell subsets within the PR3+ B cell pool differed significantly between B cell recurrence and baseline. Among total B cells, there was a relative increase of the transitional and naive subsets and a reduction of the memory subsets at B cell recurrence. Patients who experienced relapse exhibited an enrichment of the frequency of PR3+ PBs (i.e., the precursor of the cells producing PR3-ANCA) compared to patients who remained in remission. Previous data have suggested that increased frequency of total PBs during remission is related to higher risk of disease relapse in patients with granulomatosis with polyangiitis (39). In contrast, our study showed that total PBs were not different between patients who experienced relapse and patients who remained in remission at B cell recurrence, and only a small minority of total PBs were PR3+ at this stage (between 0% and 5%). Among these, it is likely that only a fraction of them will be able to terminally differentiate into plasma cells and produce high-affinity PR3-ANCA IgG. An alternative explanation for the diverging findings by von Borstel et al is that those patients received a variety of different additional conventional immunosuppressive treatments which may have affected the final results (39). For all these reasons, we believe that changes in PR3+ PBs could be more biologically meaningful than changes in total PBs in PR3-ANCA–positive AAV.

Consistently, a higher frequency of PBs within circulating autoreactive PR3+ B cells at B cell recurrence was associated with shorter time to relapse. Additionally, increased levels of PR3+ PBs were more likely to be found in patients who experienced relapse in the following 12 months, and this increase was 50% more likely to occur when compared to patients who remained in remission, strongly linking autoreactive PBs to relapse. In addition, the return of PR3-ANCA or increase in PR3-ANCA levels did not preclude the return or increase of the PR3+ PB subset. In other words, autoreactive PBs were significantly higher at B cell recurrence in patients who experienced relapse compared to patients who remained in remission, further increasing in the 12 months preceding each relapse, being detected concurrently or before an increase in PR3-ANCA titer. Because of these associations in relation to the relapse timeline, it is possible that expansion of PBs within the circulating autoreactive B cell pool may provide a source of high-affinity antibody secreting cells that promote relapses in AAV.

In patients with lupus undergoing B cell–targeted therapy with belimumab, the percentage of autoreactive naive B cells decreased from baseline during follow-up while anergic B cells seemed to increase (22,37). In patients with rheumatoid arthritis treated with methotrexate and tumor necrosis factor inhibitors, the posttreatment frequencies of autoreactive mature, naive B cells were elevated and similar to pretreatment (40). Thus, there are differences and similarities between different autoimmune diseases in their autoreactive B cell responses to different immunemodulating therapies. In contrast with methotrexate and tumor necrosis factor inhibitor–treated rheumatoid arthritis, our findings show that RTX in AAV does significantly affect the autoreactive B cell pool composition by reducing the accumulation of autoreactive naive B cells, as occurs in belimumab-treated lupus patients. Taken together, our methodology may prove useful in the future to assess restoration of tolerance in response to different therapeutic agents in AAV.

From a methodologic perspective, our customized flow cytometry method using rPR3 as a ligand has been proven to be specific for human PR3–B cell receptor (PR3-BCR) expressed by hybridoma cell lines (27). We have also shown that only the B cells from patients with PR3-AAV were able to secrete PR3-ANCA IgG as compared to MPO-AAV patients and healthy controls in PBMC cultures and enzyme-linked immunospot assay analysis (26). ELISA of culture supernatants from bulk-sorted B cells and almost 200 single cell–sorted PR3+ and PR3– immortalized B cell clones showed that the PR3+ B cell pool from PR3-AAV patients, as detected by cell surface staining with autoantigen in flow cytometry, contains antibody-secreting cells that are specific sources of PR3-ANCA. These data demonstrate that autoantigen-specific staining by FACS can accurately identify PR3-ANCA+ B cells in AAV, similar to what has been documented for other rheumatic diseases (22,41). However, in primary B cells, our FACS method may also identify a subset of B cells expressing BCRs with low affinity to PR3. To what extent lowaffinity anti-PR3 BCR+ B cell populations significantly contribute to the observed PR3+ B cell pool and produce PR3-ANCA with pathogenic potential requires future studies at the single-cell level.

One strength of our work is that it is based on the analysis of data and samples obtained during the conduct of a clinical trial, and patients included in this study underwent standardized, prolonged clinical monitoring and systematic blood sampling, and were treated with what is now considered standard of care (13). By means of the novel flow cytometry method that we used to detect autoreactive B cells in this longitudinal analysis, we were able to identify the repopulating autoreactive PR3+ B cells throughout the course of the disease, providing new insights into the mechanisms of relapse, as shown by enrichment of PBs within the autoreactive pool.

This is a discovery study that has limitations. First, the number of patients studied is small and limited to PR3-AAV, and the percentage of PR3+ PBs is derived from a post hoc analysis that requires confirmation in future longitudinal studies. A precise, longitudinal characterization of antigen-specific B cells in different samples requires a lot of effort from both individual patients and the study team. The patients included were all treated with RTX to effectively induce remission and were not receiving treatment during the follow-up according to the trial protocol, including glucocorticoids, which are known to be lymphocytolytic and to interfere with B cells (42,43), representing an exceptional model to study B cell dynamics. Second, not all the patients had the same length of follow-up, and patients who experienced a severe relapse were retreated with additional cycles of RTX, whereas patients who experienced nonsevere relapse only received glucocorticoids, according to the trial protocol.

Third, all the samples studied were cryopreserved, which could theoretically have affected our findings on autoreactive B cell subsets. However, previously published studies have indicated that cryopreserved cells can be stored for decades without general tendency toward cell loss over time (44), without significant decrease in viability, and without changes in fluorescence intensity in subsequent flow cytometry for most of the subsets (including CD19 cells) (45). Furthermore, other studies have shown the percentage and absolute numbers of freshly analyzed total PBs to be similar to those cryopreserved that we analyzed in this study (39). Finally, thanks to the use of cryopreserved samples, we were able to process all the samples under the same conditions (same flow cytometer calibration, etc.), potentially protecting the experiment from intercurrent nonbiologic variables usually referred to as batch effect.

In conclusion, our findings show the restructuring of B cell and autoreactive B cell compartments in peripheral blood in AAV after B cell depletion with successful RTX treatment and demonstrate that early changes within the autoreactive B cell pool are associated with the future outcome. Our results indicate that enrichment of PBs within the autoreactive pool after successful treatment with RTX is linked to subsequent relapse, identifying a potential mechanistic target for more durable treatment strategies in AAV.

## Supplementary Material

supinfo
