# Supplementary material for: Autoreactive Plasmablasts After B Cell Depletion With Rituximab and Relapses in Antineutrophil Cytoplasmic Antibody–Associated Vasculitis
Source: Arthritis Rheumatol. Author manuscript; Available in PMC 2024 May 1. (PMC10280646; doi:10.1002/art.42388)

**SUPPLEMENTARY MATERIALS**

**Supplementary Tables…………………….…………………………………………………2**

**Supplementary Methods………………………………………………………………........4**

**Supplementary Figures……………………………………………………………………...9**

**Supplementary Tables**

| **Supplementary Table 1. Baseline features of subjects with ANCA-associated vasculitis included in the study, by future relapse.** | | | |
| --- | --- | --- | --- |
| **Characteristics** | **Relapsers (n=10)** | **Non-relapsers (n=13)** | **p value** |
| **Age at diagnosis,** median [IQR] | 45 [27.25;54] | 54 [47,70] | 0.0761 |
| **Male sex**, % (number) | 80% (8) | 54% (7) | 0.9624 |
| **GPA,**% (number) | 100% (10) | 100% (13) | 1.000 |
| **New Disease at enrollment** (vs relapsing disease), % (number) | 40% (4) | 61% (8) | 0.4136 |
| **Any granulomatous manifestation,** % (number) | 100% (10) | 77% (10) | 0.2292 |
| **Any capillaritis’ manifestation,** % (number) | 80% (8) | 92% (12) | 0.5596 |
| **Any renal involvement,** % (number) | 50% (5) | 77% (10) | 0.2213 |
| **Glucocorticoid treatment at screening,** % (number) | 80% (8) | 77% (10) | 1.000 |
| **Baseline BVAS/WG score,**  median [IQR] | 9.5 [7; 11] | 9 [6.5; 10] | 0.777 |
| **Albumin**,g/dL,median [IQR] | 3.8[3.25;3.9] | 3.8 [3.2; 4.05] | 0.1414 |
| **CRP**, mg/L, median [IQR] | 0.8 [0.45; 2.25] | 2.8 [1.1; 6.3] | 0.3094 |
| **Baseline eGFR** mL/min/1.73 m^2^, median [IQR] | 113.4 [90.5; 156.3] | 62.2 [34.4; 107.7] | 0.0369 |
| **PR3-ANCA IgG**, IU, median [IQR] | 243.5 [95.7; 327] | 266 [86.4;366] | 0.6418 |

All the subjects were white except a Hispanic subjects in the non-relapsers group**.**

*Abbreviations: GPA=granulomatosis with polyangiitis; BVAS/WG=Birmingham Vasculitis Activity Score for Wegener's Granulomatosis; CRP= C-reactive protein; eGFR=estimated glomerular filtration rate, by means the Modification of Diet in Renal Disease (MDRD) study equation; PR3=proteinase-3; ANCA=anti-neutrophil cytoplasmic antibodies; SD=standard deviation; IQR= Interquartile range*.

| **Supplementary Table 2. Relationship between PR3-ANCA IgG titer increase and frequency of plasmablasts within PR3-reactive B cell increase.** | | | |
| --- | --- | --- | --- |
| **Characteristics** | **All (n=23)** | **Relapsers (n=10)** | **Non-relapsers (n=13)** |
| **PR3-ANCA IgG titer increase before PB-PR3 increase** | 0 | 0 | 0 |
| **PR3-ANCA IgG titer increase with PB-PR3 increase** | 13 (57%) | 7 (70%) | 6 (46%) |
| **PR3-ANCA IgG titer increase after PB-PR3 increase** | 7 (30%) | 3 (30%) | 4 (31%) |
| **PR3-ANCA IgG remain undetectable** | 3 (13%) | 0 (0%) | 3 (23%) |

All comparisons between relapsers and non relapsers were nonsignificant (p>0.05).

*PR3-ANCA IgG=Proteinase 3 ANCA Immunoglobulin G; ANCA=anti-neutrophil cytoplasmic antibodies; PB=Plasmablasts*

**Supplementary Methods**

**Supplementary Methods 1**

Trial participants were followed until the last enrolled patient had completed 18 months of follow-up, ensuring an extended follow-up for the majority of enrolled patients. The RAVE study was a multicenter, double-blind, placebo-controlled trial that showed the non-inferiority of RTX (375 mg/m^2^ intravenously each week for 4 weeks) compared to oral cyclophosphamide (2 mg/kg for 3–6 months) followed by azathioprine (AZA) (2 mg/kg, up to 150 mg/day) for the treatment of severe AAV(12).

All clinical data were obtained from the trial database. Disease activity was measured using the Birmingham Vasculitis Activity Score for Wegener’s Granulomatosis (BVAS/WG)(29). The primary outcome of the trial was defined as complete remission (BVAS/WG=0 and prednisone=0) within 6 months from randomization.

Relapse was defined as a BVAS/WG≥1 after complete remission, severe relapse was defined as BVAS/WG≥3 or the recurrence of at least one major BVAS/WG item following disease remission, requiring re-treatment with RTX.

**Supplementary Methods 2**

This variant corresponds to the mature form of the protein (deletion of the N-terminal propeptide, allowing a mature conformational state), enzymatically inactive (with the introduction of S195A point mutated to avoid the protease activity which could digest different proteins including immunoglobulins), and produced by stable transfection of HEK-293 cells(31). This rPR3 is well recognized by PR3-ANCA from patients with GPA(32). Supernatant was harvested after a 48h of culture in serum-free medium, and rPR3 was purified using a column loaded with the anti-human PR3 monoclonal antibody (Ab) MCPR3-2(25) following recommendations from the supplier (CNBr-Activated Sepharose 4 Fast Flow, GE HealthCare), concentrated, and quantified by Coomassie Plus (Pierce, Rockford, IL). We biotinylated rPR3 using a commercial biotinylation kit (Lightning-Link Rapid Biotin Conjugation Kit, Innova Biosciences, Cambridge, UK), as previously described(27).

**Supplementary Methods 3**

All cultures were done in Iscove’s modified Dulbecco’s medium (IMDM), with GlutaMax-I (Gibco/ Thermo Fisher Scientific, Waltham, MA) and further supplemented with 50 µg/ml human transferrin, 5 µg/ml insulin (both from Sigma-Aldrich, St. Louis, MO) and 10% fetal calf serum (Atlanta Biologicals, Flowery Branch, GA).

In B cell culture for ELISA, B cells were cultured for 96 hours in this media with the following cytokines: soluble CD40L (0.1 µg/ml), enhancer for ligands (1.0 µg/ml; both from Enzo Life Sciences, Farmingdale, NY), CpG-ODN 2006 (Hycult Biotechnology, Uden, The Netherlands), IL-2 (50 ng/ml), IL-10 (50 ng/ml) and IL-15 (10 ng/ml; all from Peprotech, Rocky Hill, NJ). Cells were plated at a density of 250,000 cells/ml in 96 well round bottom plate in 250μl per well. Cytospin smears of B cells harvested at day 4 were stained for qualitative assessment with May-Grünwald Giemsa, and with AMCA goat anti-human IgG 1:100 (Jackson ImmunoResearch, Ely, UK) after cell permeabilization, as previously described(34). The presence of PR3-ANCA IgG in cell culture supernatants was then assessed by an in-house direct ELISA based on reactivity against rPR3. Plates were coated with 2 ug/ml purified rPR3, culture supernatant (containing the PR3-ANCA as the antibody of interest) was added, and biotinylated goat anti-human IgG 1:5000 and streptavidin-horseradish peroxidase (HRP, DAKO) 1:10.000 were used for the detection of bound PR3-ANCA IgG. Culture supernatants were tested undiluted. Purified PR3-ANCA IgG and MPO-ANCA IgG from patients used as positive and negative controls were both diluted in RPMI growth medium at a concentration of 0.125 μg/ml. Roswell Park Memorial Institute 1640 (RPMI) growth medium was used as negative controls.

**Supplementary Methods 4**

For B-cell immortalization, peripheral blood was collected from a patient with untreated, severe PR3-AAV under a Johns Hopkins University IRB-approved research protocol(IRB00075499). PBMCs were isolated by density gradient centrifugation (Lymphoprep, Serumwerk) and cryopreserved in CryoStor CS10. To generate LCLs, cryopreserved B cells were immortalized using EBV-containing cell culture supernatants as previously described (8) and cluster generation monitored. LCLs were maintained in DMEM 1640 medium (ATCC) supplemented with 10% fetal bovine serum and 1% Penicillin-Streptomycin (Thermo Fisher). For flow cytometry and cell sorting, LCLs were stained with recombinant human PR3 (His-tagged, Abcam) and binding detected with DyLight 650-conjugated anti-His6 (Abcam). Dead cells were identified and excluded using LIVE/DEAD Fixable Violet Stain (Thermo Fisher). Staining conditions omitting rPR3, DyLight 650-conjugated anti-His6, unstained cells, and single stained cells were used as controls. PR3+ and PR3- LCLs were single cell sorted into sterile 96-well plates and maintained in culture media. Cell culture supernatants were harvested and antibody binding to native PR3 (Athens) analyzed using an in-house ELISA assay. Briefly, 96-well high-binding microplates (Costar) were coated with 100 ng/ well native PR3 in PBS 1x, pH 7.4 (Thermo Fisher) overnight at 4 degrees Celsius. Plates were washed and PR3 inactivated on the plate using 2 mM PMSF (Cell Signaling Technology) in PBS for 30 minutes at room temperature. Unspecific binding sites were blocked in 1x blocking buffer (Abcam). Coated plates were incubated with 200 uL of cell culture supernatants for 2 hours at room temperature to allow for antibody binding. Washed plates were incubated with AffiniPure HRP-conjugated goat anti-human IgM and anti-human IgG (Fc-gamma) (Jackson ImmunoResearch) for 1 hour at room temperature. Washed plates were incubated with SureBlue TMB substrate for 15 min and HRP reaction stopped. ODs were read at 450 nm and 620 nm (background). A patient-derived monoclonal IgG anti-PR3 antibody was used as a positive control. P values were determined by 2-tailed Mann-Whitney test (GraphPad Prism). P value in the figure is indicated as **** for p < 0.0001.

**REFERENCES OF THE SUPPLEMENTARY METHODS**

1. Stone JH, Merkel P a, Spiera R, Seo P, Langford C a, Hoffman GS, et al. Rituximab versus cyclophosphamide for ANCA-associated vasculitis. *N Engl J Med* 2010;363:221–32.

2. Stone JH, Hoffman GS, Merkel PA, Min YI, Uhlfelder ML, Hellmann DB, et al. A disease-specific activity index for Wegener’s granulomatosis: modification of the Birmingham Vasculitis Activity Score. International Network for the Study of the Systemic Vasculitides (INSSYS). *Arthritis Rheum* 2001;44:912–920.

3. Capizzi SA, Viss MA, Hummel AM, Fass DN, Specks U. Effects of carboxy-terminal modifications of proteinase 3 (PR3) on the recognition by PR3-ANCA. *Kidney Int* 2003.

4. Rasmussen SM, Bilgrau AE, Schmitz A, Falgreen S, Bergkvist KS, Tramm AM, et al. Stable phenotype of B-cell subsets following cryopreservation and thawing of normal human lymphocytes stored in a tissue biobank. *Cytom Part B - Clin Cytom* 2015.

5. Sun J, Fass DN, Hudson JA, Viss MA, Wieslander J, Homburger HA, et al. Capture-ELISA based on recombinant PR3 is sensitive for PR3-ANCA testing and allows detection of PR3 and PR3-ANCA/PR3 immunecomplexes. *J Immunol Methods* 1998.

6. Cornec D, Berti A, Hummel A, Peikert T, Pers JO, Specks U. Identification and phenotyping of circulating autoreactive proteinase 3-specific B cells in patients with PR3-ANCA associated vasculitis and healthy controls. *J Autoimmun* 2017;84:122–131.

7. Jourdan M, Caraux A, Vos J De, Fiol G, Larroque M, Cognot C, et al. An in vitro model of differentiation of memory B cells into plasmablasts and plasma cells including detailed phenotypic and molecular characterization. *Blood* 2009;114.

8. Penno, M.B., Pedrotti-Krueger, M. & Ray, T. Cryopreservation of whole blood and isolated lymphocytes for B-cell immortalization. Journal of Tissue Culture Methods 15, 43–47 (1993).

**Supplementary Figures**

**Supplementary Figure 1.** Gating strategy for autoreactive cell subsets within PR3^+^ B cell pool.

**
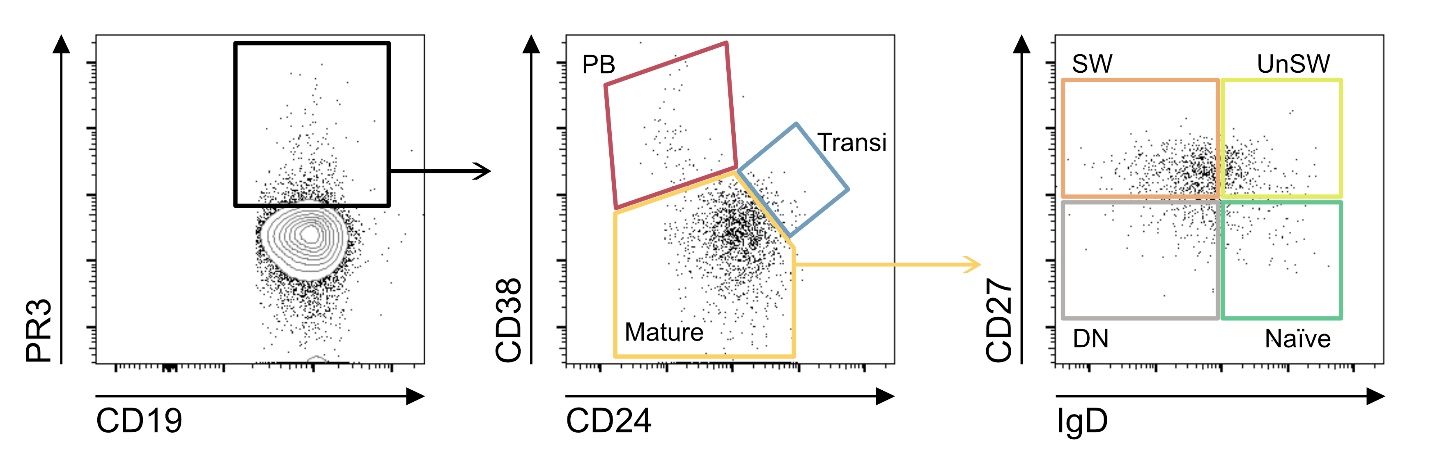
**

**Supplementary Figure 2.** Frequency at baseline of total B cells (among PBMC) **(A)** and subsets within B cells **(B)** in participants that are going to relapse (open bars) or not (black bars)**.** Frequency at baseline of total PR3^+^ B cells (among B cells) (**C**) and subsets within PR3^+^ B cells (**D**) in participants that are going to relapse (open bars) or not (black bars). PR3^+^ PBs by severe relapse (open bars) and non-severe relapses (black bars) at B cell recurrence (**E**). PR3-ANCA IgG titer at B cell recurrence in subjects that are going to severely relapse (open bars) and those that are not going to relapse (black bars) **(F).** Significant p value was reported as * when <0.05, ** when <0.01.


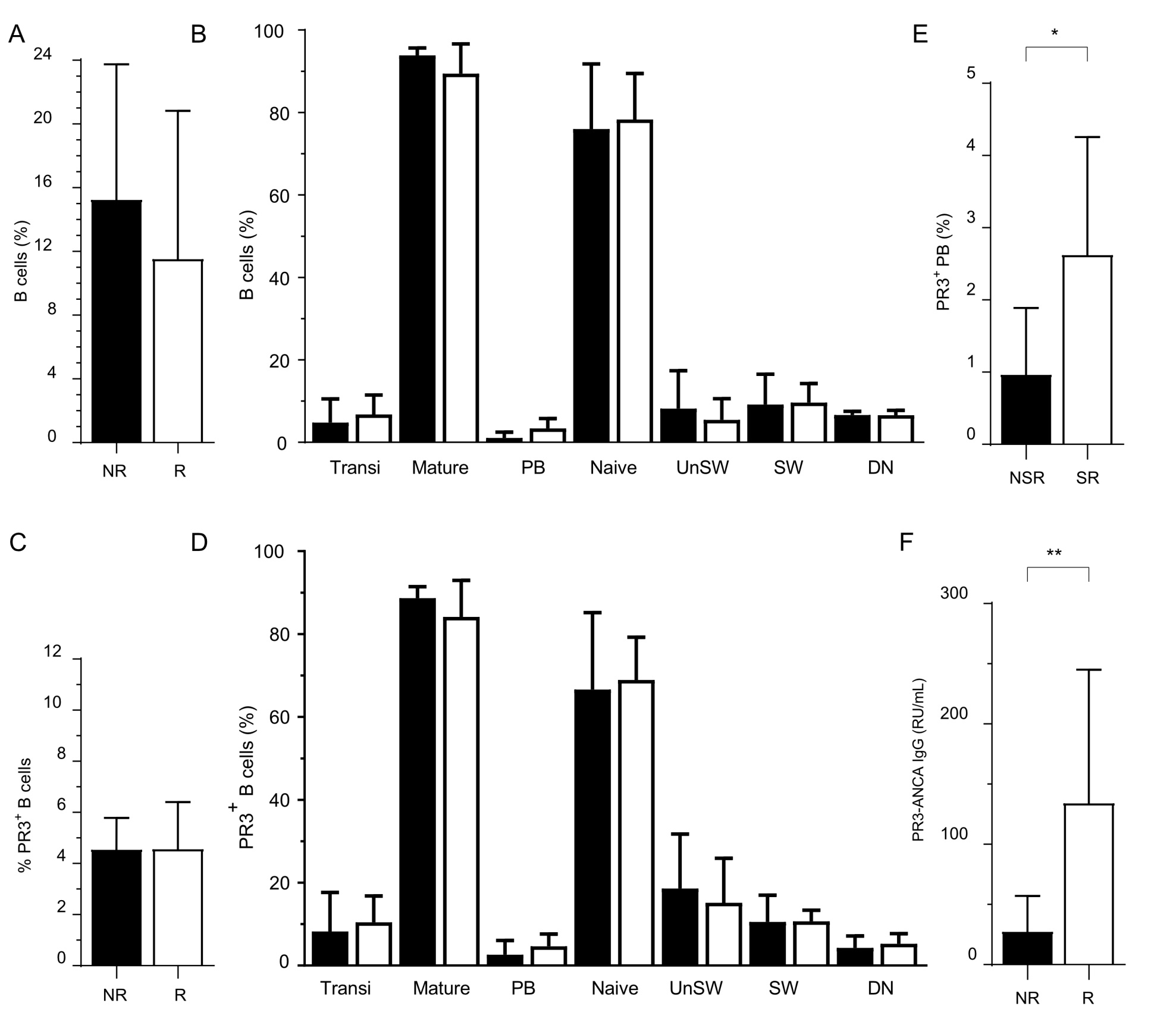


**Supplementary Figure 3**. Receiver operating characteristic curve (in red) showing the ability of PB within PR3^+^ pool to distinguish relapsing from non-relapsing patients at B cell recurrence. The Area Under the Curve (AUC) correspond to 0.79, showing that PB-PR3^+^ equal to 1.6% is the optimal cut-off to separate relapsers from long-term remitters (sensitivity=80%, specificity 77%, p=0.0129).


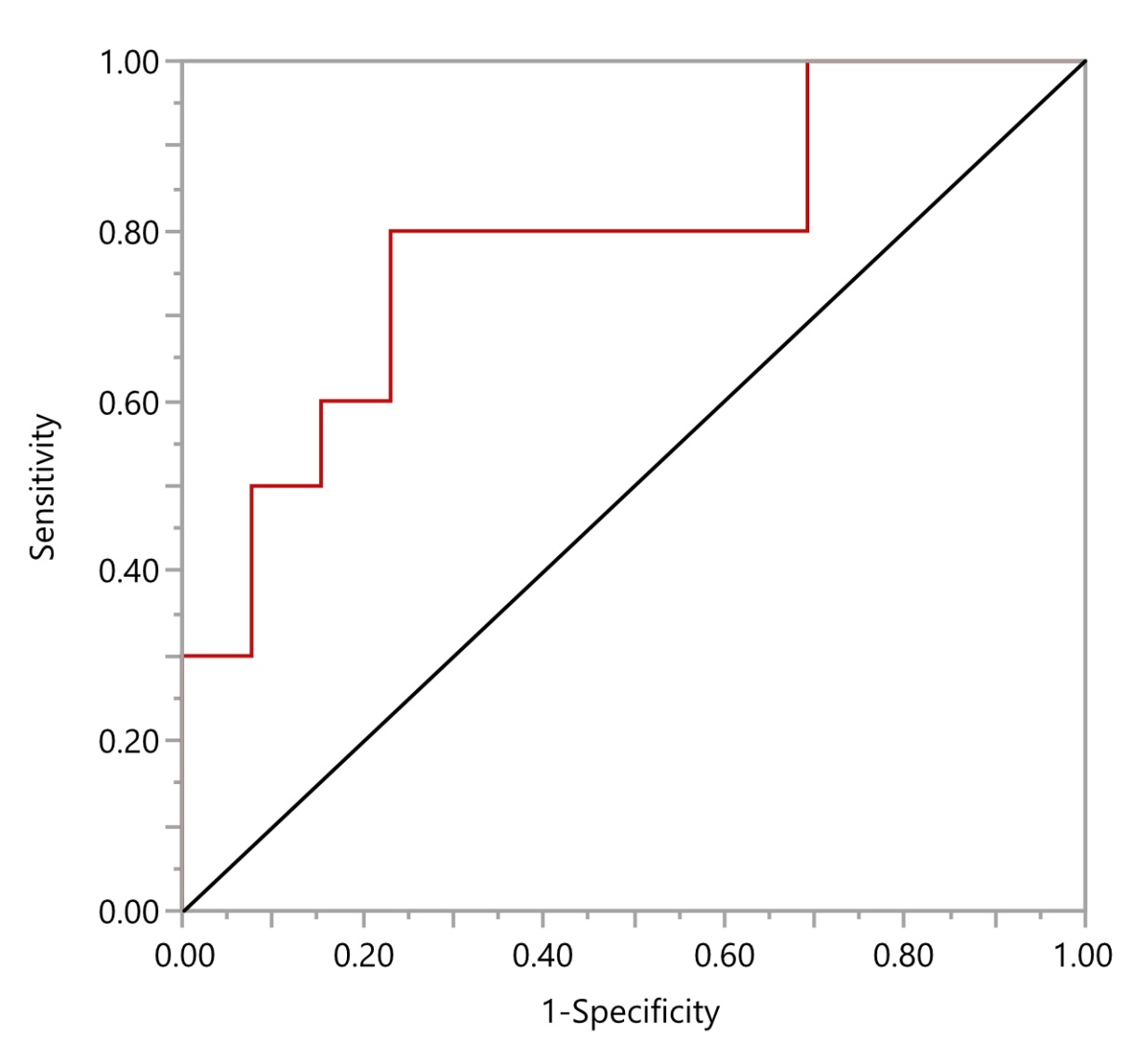

Supplement: supinfo [file NIHMS1844052-supplement-supinfo.docx]
